# Branched-Chain Amino Acids Enhance Premature Senescence through Mammalian Target of Rapamycin Complex I-Mediated Upregulation of p21 Protein

**DOI:** 10.1371/journal.pone.0080411

**Published:** 2013-11-06

**Authors:** Masayuki Nakano, Akio Nakashima, Taiki Nagano, Shintaro Ishikawa, Ushio Kikkawa, Shinji Kamada

**Affiliations:** 1 Biosignal Research Center, Organization of Advanced Science and Technology, Kobe University, Kobe, Japan; 2 Department of Biology, Graduate School of Science, Kobe University, Kobe, Japan; 3 Otsuka Pharmaceutical Co., Ltd., Minato-ku, Tokyo, Japan; Roswell Park Cancer Institute, United States of America

## Abstract

Branched-chain amino acids (BCAAs) have been applied as an oral supplementation to patients with liver cirrhosis. BCAAs not only improve nutritional status of patients but also decrease the incidence of liver cancer. Mammalian target of rapamycin (mTOR) links cellular metabolism with growth and proliferation in response to nutrients, energy, and growth factors. BCAAs, especially leucine, have been shown to regulate protein synthesis through mTOR activities. On the other hand, cellular senescence is suggested to function as tumor suppressor mechanisms, and induced by a variety of stimuli including DNA damage-inducing drugs. However, it is not clear how BCAA supplementation prevents the incidence of liver cancer in patients with cirrhosis. Here we showed that human cancer cells, HepG2 and U2OS, cultured in medium containing BCAAs with Fischer's ratio about 3, which was shown to have highest activities to synthesize and secrete of albumin, had higher activities to induce premature senescence and elevate mTORC1 activities. Furthermore, BCAAs themselves enhanced the execution of premature senescence induced by DNA damage-inducing drugs, which was effectively prevented by rapamycin. These results strongly suggested the contribution of the mTORC1 pathway to the regulation of premature senescence. Interestingly, the protein levels of p21, a p53 target and well-known gene essential for the execution of cellular senescence, were upregulated in the presence of BCAAs. These results suggested that BCAAs possibly contribute to tumor suppression by enhancing cellular senescence mediated through the mTOR signalling pathway.

## Introduction

The level of amino acids in the peripheral blood of patients with chronic liver disease is commonly changed by metabolic impairment. The branched-chain amino acids (BCAAs) are valine, leucine, and isoleucine, and the molar ratio of BCAAs to aromatic amino acids (AAAs) known as Fischer's ratio is normally 3.0 to 3.5 in plasma [1]. BCAAs serve as an important fuel source for peripheral tissues in patients with liver cirrhosis [2]. As the increase of plasma AAAs level was caused by increased degradation of muscle protein and decreased metabolism in liver, the Fisher's ratio fell typically below 2.0 in accordance with severity of liver disease. On the other hand, human serum albumin is the most abundant plasma protein, which shows about 50% of the total protein content, and patients with advanced cirrhosis have hypoalbuminemia caused by decreased synthesis in hepatocytes. The synthesis and secretion of albumin were highest when primary hepatocytes were cultured in medium with an appropriate Fischer's ratio of 3 [3]. Accordingly, the administration of an oral supplementation with BCAA granules to cirrhosis patients improved hypoalbuminemia and the prognosis [4]–[6]. In addition, hepatocellular carcinoma is commonly associated with chronic viral hepatitis and cirrhosis, and more importantly, BCAA supplemental therapy decreased the incidence of hepatocellular carcinoma in cirrhotic patients [7].

Mammalian target of rapamycin (mTOR) is activated by diverse stimuli, such as nutrients, energy, stress signals, and growth factors, to link cellular metabolism with growth and proliferation [8], [9]. mTOR forms two distinct multiprotein complexes: mTORC1 and mTORC2. mTORC1, which is sensitive to rapamycin, phosphorylates and activates p70 S6 kinase, and the kinase in turn phosphorylates ribosomal S6 protein, leading to increased protein synthesis. Activated mTORC1 also phosphorylates eIF4E-binding protein 1 (4E-BP1) and promotes the formation of the protein synthesis initiation complex. It has been shown that amino acids regulate protein synthesis through mTOR [10], and that leucine activates mTOR in the hepatic carcinoma cell lines [11]. Leucine stimulates protein synthesis in skeletal muscle and adipose tissue of food-deprived rats via a rapamycin-sensitive pathway [12], [13]. Therefore, mTORC1 is suggested to be regulated by amino acids [8], [9]. As BCAAs, especially leucine, promote the production of albumin in rat primary hepatocytes through an mTOR signal transduction system [14], BCAAs are suggested to play essential roles in metabolic disorders mediated through the mTORC1 pathway. On the other hand, mTORC2, which is neither directly or acutely sensitive to rapamycin and is generally insensitive to nutrients and energy signals, responds to growth factors such as insulin. Insulin activates mTORC2 leading to the activation of protein kinase B (PKB)/AKT. Activated PKB/AKT mediates the metabolic actions of insulin such as potentiating glucose transport and promoting mTORC1 signalling to drive protein synthesis and cell growth. It has been reported that deregulation of multiple elements of the mTOR pathway, including PKB/AKT, PI3K, 4E-BP1, eIF4E, Rheb, S6K1, LKB1, PTEN, and TSC1/TSC2, was found in many types of cancers [8], [9].

Cellular senescence was first mentioned as a state of irreversible growth arrest of normal human fibroblasts, which is termed replicative senescence because telomeres are progressively shortened by replication and ultimately causing cells to reach their “Hayflick limit” [15], [16]. Senescence can be induced by a variety conditions, such as aberrant oncogenic activation, DNA damage, and oxidative stress. This type of cellular senescence was called as premature senescence. It was suggested that DNA damage could be a common cause for various forms of senescence induced by different stimuli including telomere shortening [17], [18]. DNA damage response is initiated with the formation of foci consisting of γ-H2AX, 53BP1, NBS1, and MDC1, and leads to activation of ATM/ATR and Chk1/Chk2, which in turn phosphorylate and stabilize p53 [18]. The expression of p21 (CIP1/WAF1), one of the p53 targets, is upregulated in senescent cells [19], and overexpression of p21 could induce a senescence-like growth arrest in some cells [20]. Recently, it was suggested that senescence functions as an effective tumor suppression mechanism by preventing cell proliferation at a risk of neoplastic transformation [21]–[25].

As described above, BCAA supplementation decreases the incidence of hepatocellular carcinoma, the mTOR signalling pathway deeply contributes to tumor formation, and cellular senescence is one of the tumor suppression mechanisms. However, the relationship among BCAAs, the mTOR signalling pathway, cellular senescence, and tumor suppression has been unclear. In the present study, we have demonstrated that cells cultured in BCAA_3 medium, which have Fischer's ratio 3.12, had higher activities to induce premature senescence and elevated mTORC1 activities. Furthermore, BCAAs themselves enhanced the execution of premature senescence and upregulated p21 protein level mediated by the mTORC1 pathway. These results indicate that BCAA supplementation possibly prevents tumor formation by enhancing cellular senescence mediated through the mTOR signalling pathway.

## Materials and Methods

### Cell culture and treatment conditions

HepG2 (a human hepatocellular carcinoma line) cells were a gift from Dr. S. Shimizu [26]. U2OS (a human osteosarcoma line) cells were purchased from ATCC. HepG2 and U2OS cells were cultured in RPMI 1640 medium (Gibco Life Technologies) and Dulbecco's modified Eagle's medium (DMEM) (Wako), respectively, supplemented with 10% fetal bovine serum (FBS). PRMI-based BCAA medium containing different amounts of BCAAs summarized in Table 1 were supplemented with 10% FBS which was dialyzed against phosphate-buffered saline (PBS) to remove residual amino acids. For senescence-associated β-galactosidase (SA-β-Gal) assay and immunoblot analysis, HepG2 and U2OS cells were pre-cultured in BCAA_1 medium one day before the treatment with etoposide (Sigma) and bleomycin (Wako). Rapamycin (Calbiochem) was added to the medium 1 hour before the addition of etoposide and bleomycin.

**Table 1:** The compositions of branched-chain amino acids and aromatic amino acids in medium.

	BCAAs^a^ (mg/l)			AAAs^b^ (mg/l)		
Medium	Val	Leu	Ile	Tyr	Phe	Fischer's ratio^c^
BCAA_0	0	0	0	28.8	15	0
BCAA_1	6.25	12.5	6.25	28.8	15	0.8
BCAA_2	12.5	25	12.5	28.8	15	1.6
BCAA_3	25	50	25	28.8	15	3.12
BCAA_4	50	100	50	28.8	15	6.24
BCAA_5	100	200	100	28.8	15	12.56
RPMI	20	50	50	28.8	15	3.7

a Branched-chain amino acids (BCAAs) comprise three essential amino acids: valine (Val), leucine (Leu), and isoleucine (Ile).

b Aromatic amino acids (AAAs) contain two amino acids: tyrosine (Tyr) and phenylalanine (Phe).

c Fischer's ratio is defined as the molar ratio of BCAAs to AAAs.

### Senescence-associated β-galactosidase staining

Cells grown in 35-mm dishes or 12-well plates were washed with PBS twice, fixed with 2% formaldehyde/0.2% glutaraldehyde in PBS for 5 min at room temperature, and washed with PBS twice. After incubation with SA-β-Gal staining solution [1 mg/ml 5-bromo-4-chloro-3-indolyl-β-D-galactoside, 40 mM citric acid/sodium phosphate (pH 6.0), 5 mM potassium ferrocyanide, 5 mM potassium ferricyanide, 150 mM sodium chloride, 2 mM magnesium chloride] at 37°C for 12 to 24 hours, cells were examined under a fluorescence microscope (model BZ-8000; Keyence). Senescent cells were identified as blue-stained cells with phase contrast, and a total of 200 cells were counted in 15 random fields to determine the percentage of SA-β-Gal positive cells.

### BrdU incorporation

U2OS cells were labeled with 10 µM 5-bromo-2-deoxyuridine (BrdU, Sigma) for 3 h. For BrdU immunostaining, cells were fixed with 4% paraformaldehyde in PBS and permeabilized with 0.5% TritonX-100. DNA was hydrolyzed by exposing cells to 2 N HCl for 10 min, and then cells were incubated with anti-BrdU antibody (BD Pharmingen, 555627) in Can Get Signal immunostain Solution B (TOYOBO) overnight at 4°C followed by incubation with Alexa Fluor 488-conjugated secondary antibodies (Molecular Probes) for 1 h at room temperature. After staining nuclei with Hoechst 33258, cells were examined under fluorescence microscope.

### Antibodies

Anti-phospho-p53 (Ser15) polyclonal antibody (9284), anti-phospho-Akt (Ser473) polyclonal antibody (9271), and anti-phospho-p70 S6 kinase (Thr389) (1A5) monoclonal antibody (9206) were obtained from Cell Signaling Technology; anti-p53 (DO-1) monoclonal antibody (sc-126) and anti-p70 S6 kinase (c-18) polyclonal antibody (sc-230) were from Santa Cruz Biotechnology; anti-p21WAF1/CIP1 monoclonal antibody (K0081-3) was from Medical & Biological Laboratories; anti-Akt polyclonal antibody (559028) was from BD Pharmingen; anti-α-tubulin monoclonal antibody (T6074) was from Sigma.

### Immunoblot analysis

Cells were lysed in ice-cold lysis buffer [50 mM Tris–HCl (pH 7.5), 150 mM NaCl, 20 mM NaF, 20 mM β-glycerophosphate, 1% Nonidet P-40, 1% Sodium Lauryl Sulfate, 10 µg/mL phenylmethanesulfonyl fluoride, 5 mM EDTA, 10 µg/mL aprotinin, 10 µg/mL leupeptin]. Equal protein amounts from total cell extracts were mixed with a sample buffer containing mercaptoethanol and heated for 10 minutes at 97°C. Protein samples were separated by SDS-polyacrylamide gel electrophoresis and blotted onto Immobilon polyvinylidene difluoride membrane (Millipore). Each protein was detected using primary antibody as indicated, horseradish peroxidase-conjugated secondary antibody, and ECL detection reagent (GE Healthcare), and the intensity of each protein band was quantitated by ImageJ.

### Reverse transcription-PCR (RT-PCR)

Total RNA was isolated from HepG2 cells using the NucleoSpin® RNA II (Takara). PCR amplification was performed using specific primers for *p21*, 5′-CGACTGTGATGCGCTAATG-3′ and 5′-TCTCGGTGACAAAGTCGAAG-3′, and for *GAPDH*, 5′-CAATGACCCCTTCATTGACCT-3′ and 5′-ATGACAAGCTTCCCGTTCTC-3′. PCR products were electrophoretically separated on an agarose gel and stained with ethidium bromide for visualization.

## Results

### DNA damage-inducing drugs cause premature senescence

**Figure 1:**
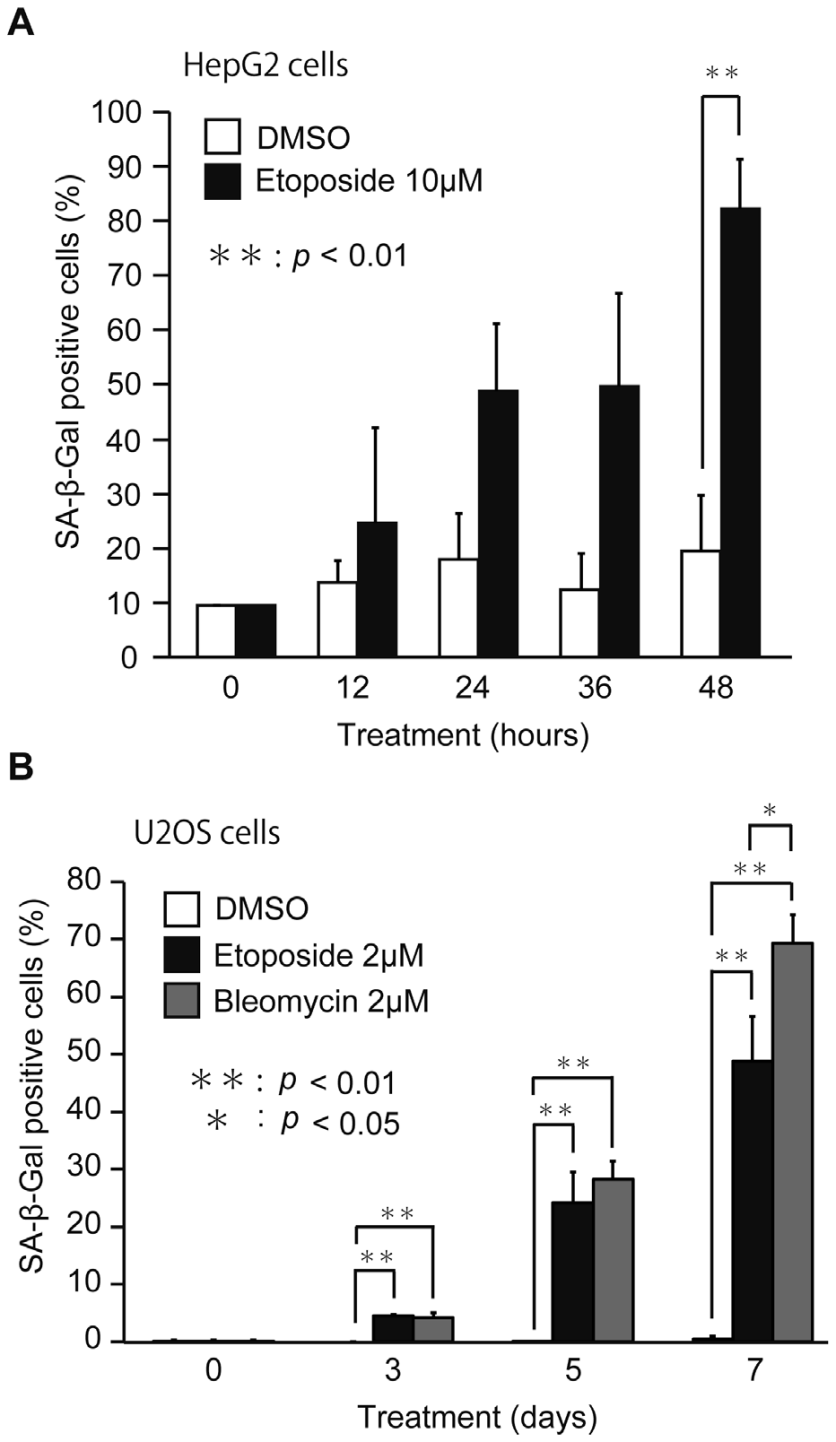
DNA damage-inducing drugs cause premature senescence. (A) HepG2 cells were cultured in RPMI medium with 0.1% DMSO or 10 µM etoposide for 0, 12, 24, 36 and 48 hours. (B) U2OS cells were cultured in RPMI medium with 0.1% DMSO, 2 µM etoposide, or 2 µM bleomycin for 0, 3, 5 and 7 days. For the assay of SA-β-Gal activity, cells stained with blue color were counted as described in Materials and Methods. The data (mean ± S.D.) were obtained from at least three independent experiments. Significant test results (*P* values) are shown.

To analyze the relationship between BCAAs and tumor formation, we used two human tumor lines, hepatocarcinoma HepG2 and osteosarcoma U2OS cells. As both cell lines have wild-type p53 gene, it is expected to normally respond to DNA damage. It has been reported that DNA damage-inducing drugs can induce senescence in tumor cells [27], [28]. Therefore, we set out to confirm whether etoposide and bleomycin induce premature senescence in HepG2 and U2OS cells (Figure 1). After treatment of HepG2 cells with 10 µM etoposide for 2 days, we observed the cells showing the arrest of proliferation and the enlargement of cell morphology (data not shown), which represent the typical features of senescent cells. As SA-β-Gal is a commonly used senescence biomarker [29], we assessed the effects of etoposide on the SA-β-Gal activity of HepG2 cells (Figure 1A). The activity of SA-β-Gal gradually increased in a time-dependent manner and about 80% of the cells showed SA-β-Gal positive within 48 hours. To confirm whether senescence is induced by etoposide, we applied the senescence assay to a human osteosarcoma cell line, U2OS (Figure 1B). Although a lower dose of etoposide, 2 µM, was enough to induce senescence, it was necessary longer periods to show the activity of SA-β-Gal. In any case, U2OS cells treated with etoposide finally represented the features of senescent cells, including cell cycle arrest, enlarged morphology, and the SA-β-Gal activity within 7 days (Figure 1B). Furthermore, another DNA damage-inducing drug bleomycin, which has a different mode of action from etoposide to induce DNA double-strand breaks, also induced premature senescence in U2OS cells (Figure 1B). These results suggested that etoposide and bleomycin could induce premature senescence in HepG2 and U2OS cells.

### Cells cultured in BCAA_3 medium have higher activities to induce premature senescence

**Figure 2:**
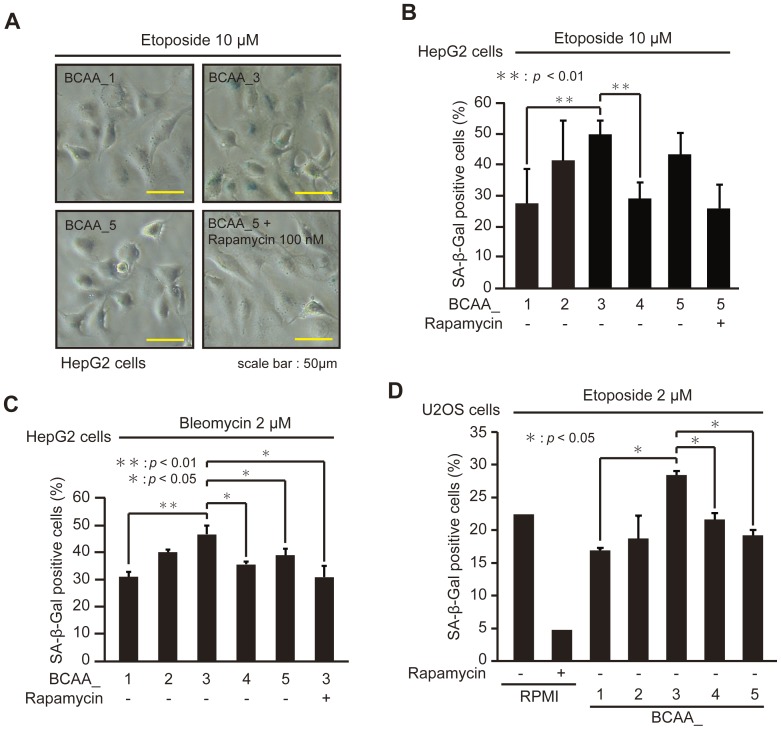
Cells cultured in BCAA_3 medium have higher activities to induce premature senescence. (A) HepG2 cells cultured in BCAA_1, 3, 5 and BCAA_5 with 100 nM rapamycin were treated with 10 µM etoposide for 2 days, and observed with microscope after SA-β-Gal staining assay. (B, C) HepG2 cells cultured in BCAA medium with or without 100 nM rapamycin as indicated were treated with 10 µM etoposide (B) or 2 µM bleomycin (C) for 2 days. (D) U2OS cells cultured in RPMI-based medium with or without 100 nM rapamycin as indicated were treated with 2 µM etoposide for 7 days. For the assay of SA-β-Gal activity, cells stained with blue color were counted as described in Materials and Methods. The data (mean ± S.D.) were obtained from at least three independent experiments. Significant test results (*P* values) are shown.

**Figure 3:**
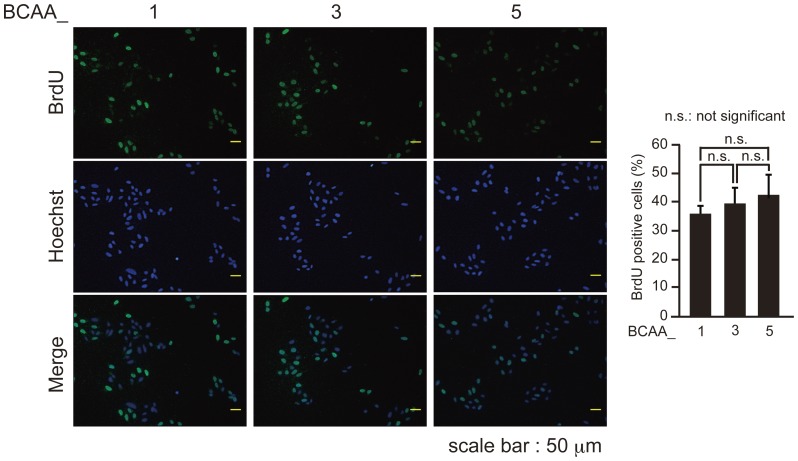
BCAA medium does not affect cell proliferation. U2OS cells cultured in BCAA medium for 7 days were labeled with 10 µM BrdU for 3 h. BrdU-labeled cells were observed with microscope after immunostaining for BrdU and Heochst staining (left), and the percentage of BrdU-positive cells was quantified (right). The data (mean ± S.D.) were obtained from at least three independent experiments. Significant test results are shown.

To examine the effects of BCAAs on the induction of premature senescence, we prepared RPMI-based medium containing various Fisher's ratio (Table 1). HepG2 cells cultured in medium with different Fischer's ratio were treated with etoposide (Figure 2A and B) and bleomycin (Figure 2C) to induce premature senescence. The ratio of SA-β-Gal positive cells was highest when cells were cultured in the medium of BCAA_3 with the Fischer's ratio of 3.12 (Figure 2A, B and C), suggesting that the induction of premature senescence of HepG2 cells induced by etoposide and bleomycin was enhanced by the medium containing BCAAs with the Fischer's ratio about 3. To confirm these results, U2OS cells cultured in the medium of BCAA_1 to BCAA_5 were treated with etoposide (Figure 2D). U2OS cells cultured in the medium of BCAA_3, in which BrdU incorporation was not significantly different from BCAA_1 and _5 (Figure 3), had the highest ratio of SA-β-Gal positive cells. These results suggested that the execution of premature senescence of HepG2 and U2OS cells induced by DNA damage-inducing drugs was enhanced by cultivation in the medium having Fisher's ratio of 3.12.

Next, we examined the effects of rapamycin, a specific mTOR inhibitor, on the enhancement of BCAAs to the execution of premature senescence, because it has been reported that BCAAs stimulate the activities of mTOR [10], [11]. The addition of rapamycin to the medium decreased the enhancement of the execution of premature senescence by BCAAs in HepG2 cells (Figure 2A, B, and C). Furthermore, the treatment of U2OS cells cultured in RPMI medium having the Fisher's ratio of 3.7 (Table 1) with rapamycin effectively prevented the execution of premature senescence induced by etoposide (Figure 2D). These results suggested that the mTOR signalling pathway contributes to the execution of premature senescence induced by DNA damage-inducing drugs.

### Cells cultured in BCAA_3 medium have higher activities of mTOR and higher protein levels of p21

**Figure 4:**
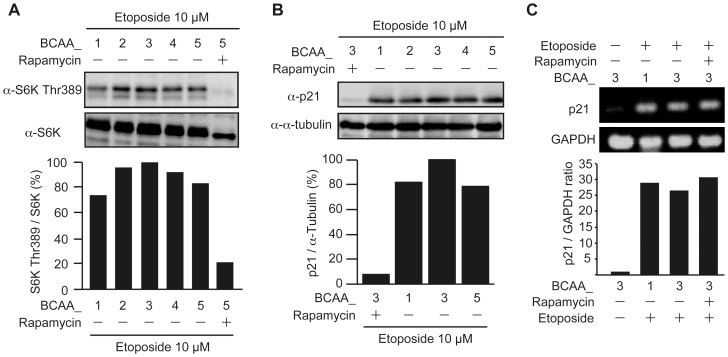
Cells cultured in BCAA_3 medium have higher activities of mTOR and higher protein levels of p21. (A) HepG2 cells cultured in BCAA medium with or without 100 nM rapamycin as indicated were treated with 10 µM etoposide for 48 hours. Cell lysates were subjected to SDS-PAGE and immunoblotted with the antibodies as indicated. The intensities of the bands corresponding to phosphorylated S6K at Thr389 and S6K were quantified by ImageJ, and the ratio of the phosphorylated S6K at Thr389 to S6K was shown as mTORC1 activities. (B) HepG2 cells cultured in BCAA medium with or without 100 nM rapamycin as indicated were treated with 10 µM etoposide for 48 hours. Cell lysates were subjected to SDS-PAGE and immunoblotted with the antibodies as indicated. The intensities of the bands corresponding to p21 and α-tubulin were quantified by ImageJ, and the ratio of p21 to α-tubulin was shown. (C) HepG2 cells cultured in BCAA medium were treated with or without 10 µM etoposide and 100 nM rapamycin as indicated for 48 hours. The mRNA expressions of *p21* and *GAPDH* were examined by RT-PCR using specific primers against *p21* and *GAPDH*. The intensities of the bands corresponding to *p21* and *GAPDH* were quantified by ImageJ, and the ratio of *p21* to *GAPDH* was shown.

To confirm whether BCAAs stimulate mTOR activities under the conditions in which cells were treated with etoposide to induce premature senescence, the phosphorylation of S6K at Thr389, a mTORC1 substrate, was assessed (Figure 4A). Although S6K Thr389 phosphorylation was observed in cells cultured in the medium of BCAA_1 through BCAA_5, the phosphorylation levels were maximum in BCAA_3 and the phosphorylation was suppressed by rapamycin, suggesting that mTORC1 was activated under these conditions and had the highest activity in BCAA_3 medium. As it was reported that mTORC1 stimulates protein synthesis [8], [9] and p21, a cyclin-dependent kinase inhibitor, can mediate cellular senescence [19], [20], the expression level of p21 protein was assessed in cells cultured with each BCAA medium after treatment with etoposide (Figure 4B). Although p21 protein was detected in cells cultured by BCAA_1 through BCAA_5, because *p21* is a DNA damage responsive gene, the protein level of p21 in BCAA_3 medium was higher than that in other BCAA medium. Furthermore, p21 protein was markedly decreased in the presence of rapamycin even in the presence of etoposide, indicating that the expression level of p21 was regulated through the mTORC1 pathway. To confirm whether the upregulation of p21 protein is mediated by translation but not transcription, the levels of *p21* mRNA were compared (Figure 4C). mRNA level for *p21* were dramatically increased after treatment with etoposide, consistent with the previous reports that the transcription of *p21* was induced by genotoxic stresses [30], [31]. However, the similar levels of *p21* mRNA were observed in BCAA_1 and BCAA_3, and more importantly rapamycin did not affect the transcription of *p21*. These results suggested that the enhancement of cellular senescence cultured in BCAA_3 medium is mediated by the upregulation of p21 protein through the mTORC1 pathway.

### BCAAs enhance the execution of premature senescence induced by DNA damage-inducing drugs

**Figure 5:**
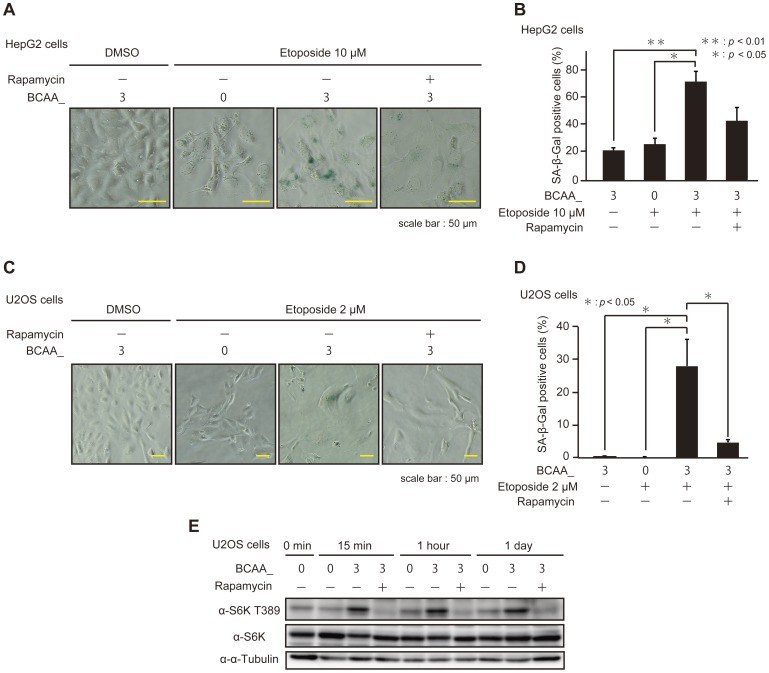
BCAAs enhance the execution of premature senescence induced by DNA damage-inducing drugs. (A) HepG2 cells cultured in BCAA medium were treated with or without 10 µM etoposide and 100 nM rapamycin as indicated for 48 hours, and observed with microscope after SA-β-Gal staining assay. (B) HepG2 cells were cultured in BCAA as described in A. For the assay of SA-β-Gal activity, cells stained with blue color were counted as described in Materials and Methods. The data (mean ± S.D.) were obtained from at least three independent experiments. Significant test results (*P* values) are shown. (C) U2OS cells cultured in BCAA medium were treated with or without 2 µM etoposide and 100 nM rapamycin as indicated for 7 days, and observed with microscope after SA-β-Gal staining assay. (D) U2OS cells were cultured in BCAA medium as described in C. The assay of SA-β-Gal activity was carried out as described in B. (E) U2OS cells cultured in BCAA medium were treated with or without 100 nM rapamycin as indicated for 24 hours and cells were harvested at each time point. Cell lysates were subjected to SDS-PAGE and immunoblotted with the antibodies as indicated.

As described above, cells cultured in BCAA_3 medium had higher activities to execute premature senescence mediated by mTOR as compared with cells cultured in BCAA_1, 2, 4, and 5. The differences, however, were not very high and it is not clear whether the effects were caused by BCAAs, because every medium contained various amounts of BCAAs that could activate mTOR at least as basal levels. Therefore, we examined the activities of BCAAs to enhance the execution of premature senescence and to stimulate mTOR kinase activities as compared cells cultured in BCAA_3 with those in BCAA_0 which contained no BCAAs (Figure 5 and Table 1). HepG2 cells cultured in BCAA_0 and BCAA_3 were treated with etoposide to induce premature senescence and assessed the SA-β-Gal activity (Figure 5A and B). Interestingly, the activity to induce premature senescence in cells cultured in BCAA_0 was significantly reduced as compared with those in BCAA_3, and the activity was suppressed by rapamycin. Similar results were obtained by using U2OS cells (Figure 5C and D). These results strongly suggested that BCAAs and mTORC1 contributed to the execution of premature senescence induced by DNA damage-inducing drugs.

To confirm whether mTORC1 was activated in cells cultured in BCAA_3 but not in BCAA_0 under the conditions in which DNA damage-inducing drugs were excluded in order to avoid the effect of DNA damage response on mTOR activities, the phosphorylation of S6K at Thr389 was assessed (Figure 5E). A faint band corresponding to S6K Thr389 phosphorylation was detected in BCAA_0 medium at 0 min, and the band was detected even at 1 day, suggesting that the weak activities of mTORC1 were sustained despite the absence of BCAAs. As the phosphorylation of S6K at Thr389 was elevated in cells cultured in BCAA_3 medium and effectively suppressed by rapamycin, the activities of mTORC1 were apparently higher in BCAA_3 than those in BCAA_0. Collectively, these results indicated that BCAAs themselves elevate the activities of mTORC1 in cells.

### BCAAs upregulate p21 protein level mediated through the mTORC1 pathway

**Figure 6:**
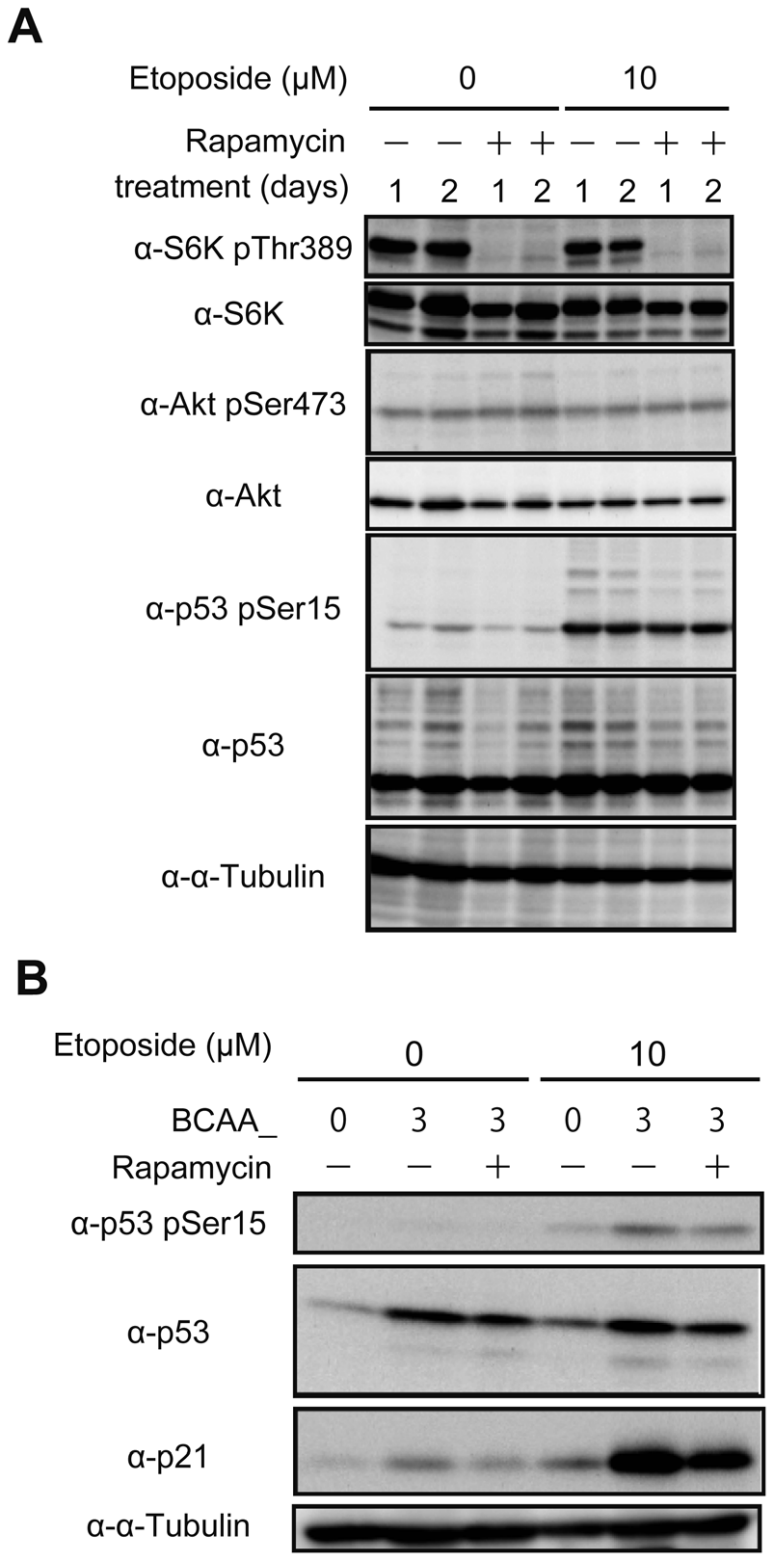
BCAAs upregulate p21 protein level mediated through the mTORC1 pathway. (A) HepG2 cells cultured in RPMI medium were treated with or without 10 µM etoposide and 100 nM rapamycin as indicated for 1 or 2 days. Cell lysates were subjected to SDS-PAGE and immunoblotted with the antibodies as indicated. (B) HepG2 cells cultured in BCAA medium were treated with or without 10 µM etoposide and 100 nM rapamycin as indicated for 2 days. Cell lysates were subjected to SDS-PAGE and immunoblotted with the antibodies as indicated.

Next we examined the relationship between DNA damage response and mTOR activities (Figure 6). HepG2 cells cultured in normal RPMI medium containing BCAAs with the Fisher's ratio of 3.7 (Table 1) were treated with or without etoposide, and the activities of mTORC1 and mTORC2 were assessed as judged by S6K phosphorylation at Thr389 as an mTORC1 substrate and Akt phosphorylation at Ser473 as an mTORC2 substrate, respectively (Figure 6A). The phosphorylated bands of S6K at Thr389 were detected and disappeared by rapamycin treatment, whereas the phosphorylation of Akt at Ser473 was observed with lower levels which was resistant to rapamycin. However, both mTORC1 and mTORC2 activities were not affected by the treatment with etoposide, suggesting that the mTOR pathways themselves were not involved in DNA damage response. In contrast, the phosphorylation of p53 at Ser15, which was suggested to response to various DNA damage agents [32], was elevated after treatment with etoposide, indicating that DNA damage response normally proceeded to activate p53, and the phosphorylation was not influenced by rapamycin. These results suggested that the site of action of mTORC1 in DNA damage response under these conditions resided downstream of p53.

Among the p53 target genes, *p21* is a well-known gene essential for the execution of premature senescence, and the protein level of p21 was higher in cells cultured in BCAA_3 than those in other BCAA-containing medium (Figure 4C). Therefore, we set out to examine the effects of BCAAs themselves on the protein levels of p21 as compared cells cultured in BCAA_3 with those in BCAA_0 which contained no BCAAs (Figure 6B). The protein levels of p53 were unchanged in cells treated with or without etoposide, while p53 in cells cultured in BCAA_3 was higher than that in BCAA_0, which may be induced by upregulated translation in the presence of BCAAs. On the other hand, the phosphorylation of p53 at Ser15, which was expected to elevate transcriptional activities, was detected only in cells treated with etoposide and the phosphorylation levels of p53 appeared to be unchanged because those were in parallel with their protein levels even though the presence of rapamycin. These results suggested that p53 was largely unaffected by the mTORC1 pathway, consistent with the results in Figure 6A. On the contrary, p21 protein level was strongly upregulated by DNA damage only in the presence of BCAAs, and the elevation of p21 protein was suppressed by rapamycin. Taken together, these results indicate that BCAAs positively regulate premature senescence by upregulating p21 protein through the mTORC1 pathway.

## Discussion

In the present study, we evaluated the effects of BCAAs on the execution of premature senescence induced by DNA damage response. The results showed that cells cultured in medium containing BCAAs having Fisher's ratio 3.12 possessed higher activities to induce premature senescence. As mTORC1 was activated and p21 was upregulated by BCAAs themselves, the execution of premature senescence induced by DNA damage-inducing drugs seemed to be enhanced by BCAAs mediated through the mTORC1 signalling pathways.

As several tumor suppressors, such as p53, p21, p16, Arf, and pRB, function as regulators of senescence, it has been suggested that senescence acts as an important tumor suppression mechanism [33], [34]. In addition, most of human cancer cells acquired the ability to proliferate permanently through reactivation of telomerase [35], suggesting a connection between telomere checkpoint and tumor suppression. Although ectopic expression of human telomerase reverse transcriptase (hTERT) in normal human cells sufficed to immortalize the cells and enhanced the ability to induce neoplastic transformation [36], [37], and transgenic mice overexpressing TERT were prone to tumorigenesis [38], [39], inhibition of telomerase in cancer cells limited proliferation through telomere shortening and cell death [40], [41]. Furthermore, it was indicated that senescence induced by telomere shortening was an effective tumor suppression mechanism *in vivo* [21], [22]. Additionally, senescent cells were found in premalignant lesions or benign tissues induced by different oncogene activation or tumor suppressor inactivation, but not in malignant tumors [23]–[25]. These results suggest that cellular senescence is a powerful tumor suppression mechanism by limiting cell proliferation, and provides an attractive therapeutic option for cancer treatments if it can be induced in tumor cells. According to this theory, cancer therapies to induce senescence by inhibiting telomerase have been tried [42]. In addition to the strategy of inhibiting telomerase as therapeutic targets, conventional chemotherapeutic drugs, which cause DNA damage, can induce senescence in various types of tumor cells in culture and *in vivo* [27], [28]. Consistent with these observations, we showed that etoposide and bleomycin, chemotherapeutic drugs causing DNA damage, induced senescence in human tumor lines, HepG2 and U2OS cells. More importantly, the treatment of chemotherapeutic drugs in combination with BCAAs enhanced the execution of premature senescence. As senescent cells were detected in human tumors after chemotherapy [27], BCAA supplementation in chemotherapy may be useful for improving therapeutic efficacy. Furthermore, as telomere shortening induces senescence mediating DNA damage [17], [18], BCAA supplementation may also be applicable to cancer therapy by inhibiting telomerase.

A low Fischer's ratio is a physiological hallmark of liver cirrhosis, and BCAA supplementation was originally devised to normalize amino acid profiles and nutritional status of the patients. Recent studies have revealed that BCAA supplementation improves not only nutritional status but also prognosis and quality of life in patients with liver cirrhosis. Furthermore, BCAA supplementation has been suggested to prevent the incidence of hepatocellular carcinoma in patients with liver cirrhosis [5]–[7]. One possible explanation for the molecular mechanisms of BCAAs to prevent cancer incidence was reported, in which BCAAs inhibited insulin-induced hepatic tumor cell proliferation by inducing apoptosis through the activities of mTORC1 and mTORC2 [43]. In addition, we demonstrated here another possible mechanism of BCAA supplementation for inhibiting cancer incidence. As BCAAs can enhance the execution of premature senescence mediated though mTORC1 activity to upregulate p21 protein, the prevention of the incidence of hepatocellular carcinoma in liver cirrhosis by BCAA supplementation may be carried out several different mechanisms including the induction of apoptosis and senescence.

It has been suggested that p53 is an attractive target to induce senescence in cancer cells, because p53 is a central player in the execution of senescence and is commonly mutated in cancer cells. Although various approaches have been tried to target p53 in order to recover normal p53 function in cancer cells, in most cases apoptosis is the prominent response responsible for tumor suppression. However, it was reported that senescence functions as a tumor suppression mechanism after restoration of p53 [44], [45]. Here we showed that BCAAs enhanced senescence induced by DNA damage mediated through the mTORC1 pathway to upregulate the translation of p21. The expression of p21 was upregulated in senescent cells and overexpression of p21 could induce features of the senescent phenotype [19], [20], while it was also suggested that the expression of p21 was not required for senescence [46]. Therefore, p53 may regulate cellular senescence induced by DNA damage at least in part by upregulating the transcription of *p21*. As the regulation of protein syntheses mediated by the mTOR pathway is expected to affect a wide variety of genes, it will be important to identify genes essential for senescence, whose transcription and translation are regulated by p53 and mTOR, respectively.
